# Maize VKS1 Regulates Mitosis and Cytokinesis During Early Endosperm Development

**DOI:** 10.1105/tpc.18.00966

**Published:** 2019-04-08

**Authors:** Yongcai Huang, Haihai Wang, Xing Huang, Qiong Wang, Jiechen Wang, Dong An, Jiqin Li, Wenqin Wang, Yongrui Wu

**Affiliations:** a National Key Laboratory of Plant Molecular Genetics, Chinese Academy of Sciences Center for Excellence in Molecular Plant Sciences, Institute of Plant Physiology and Ecology, Shanghai Institutes for Biological Sciences, Chinese Academy of Sciences, Shanghai 200032, China; b University of the Chinese Academy of Sciences, Beijing 100049, China; c School of Agriculture and Biology, Shanghai Jiao Tong University, Shanghai 200240, China.

## Abstract

Cell number is a critical factor that determines kernel size in maize (*Zea mays*). Rapid mitotic divisions in early endosperm development produce most of the cells that make up the starchy endosperm; however, the mechanisms underlying early endosperm development remain largely unknown. We isolated a maize mutant that shows a varied-kernel-size phenotype (*vks1*). *Vks1* encodes ZmKIN11, which belongs to the kinesin-14 subfamily and is predominantly expressed in early endosperm development. VKS1 dynamically localizes to the nucleus and microtubules and plays key roles in the migration of free nuclei in the coenocyte as well as in mitosis and cytokinesis in early mitotic divisions. Absence of VKS1 has relatively minor effects on plants but causes deformities in spindle assembly, sister chromatid separation, and phragmoplast formation in early endosperm development, thereby resulting in reduced cell proliferation. Severities of aberrant mitosis and cytokinesis within individual *vks1* endosperms differ, thereby resulting in varied kernel sizes. Our discovery highlights VKS1 as a central regulator of mitosis in early maize endosperm development and provides a potential approach for future yield improvement.

## Abstract (precis)

A kinesin-14 motor encoded by *Vks1* regulates mitosis and cytokinesis during early maize endosperm development and deformities within *vks1* endosperms differ, thereby resulting in varied kernel sizes.

## INTRODUCTION

Flowering plants have evolved an advanced mode of reproduction known as double fertilization. A pollen grain bears two genetically identical haploid sperms, of which one fertilizes the egg to form a diploid zygote that eventually develops into the adult plant body; the other fuses with the two polar nuclei in the central cell and grows into the triploid endosperm, a nutritive storage organ characteristic of angiosperm seeds (Hamamura et al., 2012). The evolutionary advantage of the endosperm could lie in the fact that both parents contribute to endosperm formation, and the plant does not invest energy in seed nutritive tissue unless the egg is successfully fertilized. The endosperm nucleus divides rapidly, and rapid storage organ formation has obvious advantages. The endosperm nourishes the embryo as it develops, when it germinates, and as the seedling grows. In cereal grains, the endosperm stores large amounts of carbohydrates and proteins, and consequently it is an important source of food, feed, and industrial raw materials.

In maize (*Zea mays*), the duration of endosperm development from pollination to seed maturity is approximately 7 weeks. Based on distinct cytological activities occurring during this process, endosperm development is divided into five stages: (1) coenocyte, (2) cellularization, (3) cell mitotic division and differentiation, (4) endoduplication and cell expansion, and (5) maturation followed by programmed cell death (PCD). The coenocytic stage is characterized by rapid and repeated divisions of the primary endosperm nucleus without cytokinesis. Two days after pollination (DAP), the individual nuclei are embedded in a thin layer of peripheral cytoplasm that surrounds a large vacuole (Brown et al., 2003; Olsen, 2004). Cellularization of the coenocyte is initiated via the formation of radial microtubule systems (RMSs) around neighboring nuclei, which create zones between them. The RMS acts as a primitive phragmoplast to form tube-like cell wall structures around each endosperm nucleus, with an opening facing inward toward the center of the endosperm. After the alveolar layer is formed, cells undergo rapid periclinal division accompanied by cytokinesis, producing an outer layer of cells and an inner layer of alveoli. After several rounds of mitosis and cytokinesis in the alveolar layer, the endosperm eventually becomes fully cellularized by 4 DAP (Brown et al., 1994; Staehelin and Hepler, 1996; Sylvester, 2000; Olsen, 2001). Then, these cells begin to undergo rapid proliferation by mitotic cell divisions that peak around 8 DAP.

Early endosperm development usually encompasses the period from 0 to 8 DAP. The mid and late endosperm development phases manifest cell differentiation, synthesis of storage reserves, and PCD and desiccation, respectively. Although the period of early endosperm development is much shorter than the mid and late phases, the coenocytic and cellularization stages are critical steps in endosperm formation in cereals and many other species. The early mitotic divisions give rise to most of the cells comprising the starchy endosperm, which is important for grain filling (Sabelli and Larkins, 2009a, 2009b; Sabelli et al., 2013, 2018). Because endosperm cell number is a major determinant of seed size and weight, a better understanding of early endosperm development is potentially critical to increasing cereal productivity.

Kinesins are a class of motor proteins that move along microtubule (MT) filaments and are powered by hydrolysis of ATP (Vale et al., 1985). All kinesin proteins have a conserved motor domain that possesses catalytic ATPase activity (Sablin et al., 1996), whereas various functions of different kinesins are conferred by divergent motor-flanking regions that are important for isoform-specific functions (Vale and Goldstein, 1990), including MT-based motility (Vale et al., 1985), intracellular transport (Hirokawa, 1998; Vale, 2003; Hirokawa et al., 2009), MT and chromosome dynamics (Heald et al., 1996; Heald, 2000), and other diverse functions.

The kinesin superfamily is divided into 14 distinct subfamilies (Lawrence et al., 2004). The kinesin-1 to kinesin-12 subfamilies are plus-end-directed motors, with the motor domain at the N terminus, whereas the kinesin-13 members are MT depolymerases, with the motor domain flanked by additional regions on both sides. The kinesin-14 subfamily members possess a unique motor domain that is located at the C terminus and is minus-end directed (Hirokawa, 1998). Plants contain a large and diverse kinesin-14 subfamily. Due to the lack of cytoplasmic dyneins in plants, the kinesin-14 proteins are thought to have evolved to fill the function of dyneins found in fungi and animals (Lawrence et al., 2001). The kinesin-14 subfamily is greatly expanded in plants, with 20 members in Arabidopsis (*Arabidopsis thaliana*), 16 in rice (*Oryza sativa*), and 21 in maize (Supplemental Figure 1; Supplemental File), whereas only four are found in the human genome (Richardson et al., 2006). Plant kinesin-14 motors are involved in spindle morphogenesis, MT-based trafficking, chromosome segregation, chloroplast distribution, and plant-specific phragmoplast formation (Lee et al., 2015; She and Yang, 2017; Gicking et al., 2018). Arabidopsis ATK1, the first kinesin-14 protein discovered in plants, primarily functions in spindle morphogenesis and chromosome segregation during meiosis (Chen et al., 2002; Marcus et al., 2002), whereas Arabidopsis ATK5 is required for mitotic spindle pole formation (Ambrose and Cyr, 2007). Plant kinesin-14 motors have also evolved novel functions. Kinesins with a calponin homology domain (OsKCH2) in rice displays processing minus-end-directed motility on single MTs as individual homodimers (Tseng et al., 2018). The plant-unique kinesin-14 member Kinesin-like calmodulin-binding protein (KCBP) plays an important role in cytoskeletal regulation of trichome cell shape (Tian et al., 2015). Recently, a highly specialized kinesin-14 motor (named KINDR) encoded on maize abnormal chromosome 10 (Ab10) was shown to localize to heterochromatic knobs and form mobile neocentromeres. KINDR can move neocentromeres along spindles to create meiotic drive, leading chromosomes to be preferentially transmitted to egg cells (Dawe et al., 2018). This finding explained the longstanding mystery of the meiotic drive of Ab10 (Schroeder and Malik, 2018). Another classic kinesin-14 member in maize is Divergent Spindle-1 (DV1), which is known to function in meiotic spindle pole organization (Higgins et al., 2016). Although the plant kinesin-14 subfamily contains many members (Vale and Goldstein, 1990), most of their molecular functions and biological roles in development remain to be elucidated.

Several signaling pathways regulating seed size have been studied in Arabidopsis and rice, but little is known about the mechanisms that regulate early endosperm development (Li and Li, 2016). Here, we report the identification of a novel mutant, *varied kernel size* (*vks1*), which exhibits nonuniform kernel sizes on a genetically homozygous ear. *Vks1* encodes ZmKIN11, a homolog of KINDR, that also belongs to the kinesin-14 subfamily. *Vks1* is most highly expressed during early endosperm development and is abundant at coenocytic and cellularization stages. VKS1 contains an N-terminal region specific for nuclear localization in interphase. When mitosis begins, VKS1 always colocalizes with MTs that function in spindle assembly, chromosome separation, and phragmoplast formation. Mutation of *vks1* results in defects in nuclear division and cytokinesis in early endosperm development and, as a consequence, leads to varied and small kernel sizes at seed maturity.

## RESULTS

### *vks1* Displays a Nonuniform Small-Kernel Phenotype

To study endosperm development, we treated the A619 inbred line with ethyl methanesulfonate (EMS), which generated a large collection of mutants, including many with visible endosperm defects. Among them, *vks1* seeds exhibited a phenotype wherein the kernel size was not only reduced but also varied greatly among individuals. *vks1* appears to contradict what is known about traditional single-gene mutations, wherein mutants usually display a qualitative and uniform alteration in phenotype.

**Figure 1.**
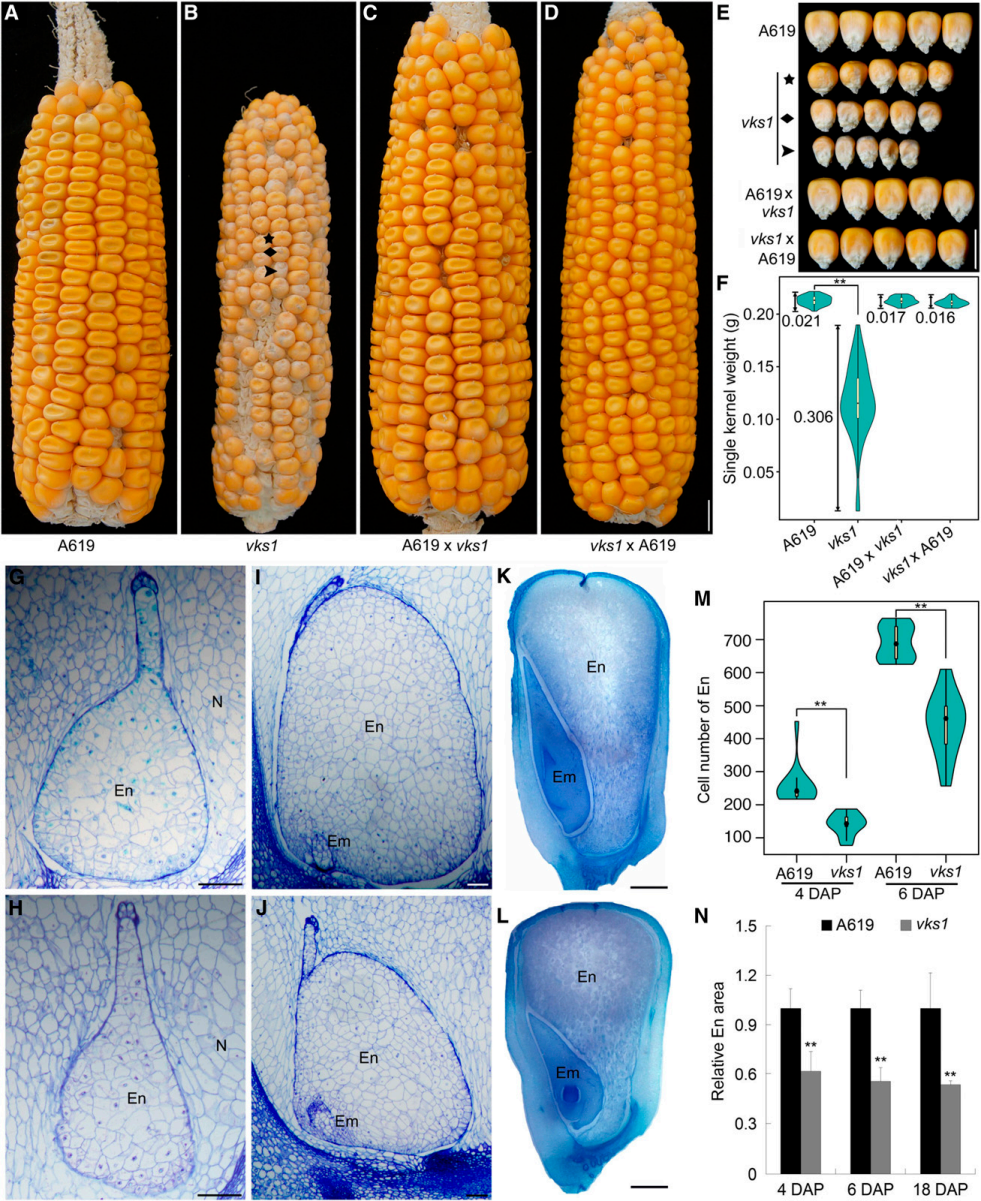
Nonuniform Small-Kernel Phenotype of *vks1*. **(A)** to **(D)** Ear phenotypes of A619, *vks1*, A619 × *vks1*, and *vks1* × A619. Three mutant seeds on a homozygous *vks1* ear representing mild, medium, and severe developmental phenotypes are indicated by an asterisk, a rhombus, and an arrowhead, respectively. Bar = 1 cm. **(E)** Seed phenotypes of A619, *vks1*, A619 × *vks1*, and *vks1* × A619. The *vks1* seeds from **(B)** with mild, medium, and severe phenotypes are indicated by an asterisk, a rhombus, and an arrowhead, respectively. Bar = 1 cm. **(F)** Seed weight variation of single kernels in A619, *vks1*, A619 × *vks1*, and *vks1* × A619. The number beside each column indicates the coefficient of variation. **, P < 0.01 as determined by Student’s *t* test. **(G)** and **(H)** Semithin sections of A619 **(G)** and *vks1* **(H)** endosperms at 4 DAP. Bars = 100 μm. Em, Embryo; En, Endosperm; N, Nucellus. **(I)** and **(J)** Semithin sections of A619 **(I)** and *vks1* **(J)** endosperms at 6 DAP. Bars = 100 μm.Em, Embryo; En, Endosperm. **(K)** and **(L)** Sections of A619 **(K)** and *vks1* **(L)** endosperms at 18 DAP. Bars = 1 mm.Em, Embryo; En, Endosperm. **(M)** Cell numbers of A619 and *vks1* endosperms at 4 and 6 DAP. **, P < 0.01 asd determined by Student’s *t* test. **(N)** Relative endosperm area at 4, 6, and 18 DAP. The ratio of the area of the endosperm to that of the whole seed in A619 and *vks1* is shown The ratio in A619 was set as 1. **, P < 0.01 as determined by Student’s *t* test.

Since EMS can produce many lesions in the genome, phenotypic variation can result from different combinations of multiple unlinked mutant genetic loci. To investigate this possibility, *vks1* was recurrently backcrossed to A619 for six generations and then self-pollinated for two generations (see Methods). The contrasting phenotypes of wild-type and *vks1* ears are compared in Figures 1A and 1B. *vks1* seeds were remarkably smaller than wild-type seeds and showed apparent variation in size. Wild-type seeds at the base of the ear are always larger than those at the tip end, but most in between appear nearly uniform in size (Figure 1A). In *vks1*, the mutant seeds could be roughly classified into three ranks with mild, moderate, and severe phenotypes (Figures 1B and 1E). Consistent with the size variation, the 100-kernel weight of *vks1* (11.63 g) was dramatically reduced compared with the wild type (29.65 g). When measured, the weight of single *vks1* seeds showed a significantly greater coefficient of variation than that of the wild type (0.306 versus 0.021; Figure 1F). When reciprocal crosses between the wild type and *vks1* were made, the resulting ears showed normal and uniform seed phenotypes (Figures 1C to 1F), consistent with *vks1* being a recessive mutation.

In the anthocyanin regulatory and biosynthetic pathways of maize, *C1* (*colored aleurone1*) and *Bz1* (*bronze1*) encode a MYB transcription factor and a uridine diphosphate glucose-flavonol 3-*O*-glucosyl transferase enzyme, respectively, both of which are required for anthocyanin synthesis. A619 is a typical yellow dent maize inbred line that bears a mutant *c1* gene and a normal *Bz1* gene, whereas W22 has the complementary genotype (normal *C1* and the mutant *bz1-mum9* allele). We mixed equal amounts of *vks1* (*vks1*;*c1*;*Bz1*) and W22 (*Vks1*;*C1*;*bz1-mum9*) pollen and applied the mixture to homozygous *vks1* plants. The seed genotypes on the resulting ears were easy to characterize by observing anthocyanin pigmentation (Supplemental Figure 2A). The purple seeds (indicative of the cross between *vks1* and W22) that were heterozygous for the three genes displayed large, uniform kernels, whereas the yellow seeds (indicative of selfing of *vks1*) consistently showed a phenotype with varied size and weight (Supplemental Figures 2B and 2C).

To further characterize the reduction in seed size, we investigated endosperm development (Figures 1G to 1N). Four and six DAP stages represented time points when cellularization was complete and early mitotic divisions are rapid, respectively. In *vks1* and the wild type, the endosperms were surrounded by the nucellus at both stages. In *vks1*, the endosperms were morphologically normal but smaller than in the wild type (Figures 1G to 1J). There were significantly fewer endosperm cells in *vks1* than in the wild type (Figure 1M), indicating that cell division during early endosperm development in *vks1* proceeded more slowly than in the wild type. At 18 DAP, when storage reserves were actively synthesized, both the seed and endosperm areas in *vks1* were smaller compared with the wild type (Figures 1K, 1L, and 1N). Light microscopy observations clearly demonstrated that starchy endosperm cells in *vks1* and the wild type were fully filled with starch granules (Supplemental Figures 3A and 3B), and the accumulation of zein and nonzein storage proteins and total starch content exhibited no discernible difference between them (Supplemental Figures 3C and 3D). Taken together, these results indicate that seed size reduction in *vks1* relates to a decrease in cell number that may occur early rather than late in endosperm development.

In addition to defects in endosperm development, the *vks1* mutant displayed abnormalities in plant development. Although *vks1* seeds of different sizes germinated normally, the resulting plants were shorter than those of the wild type (Supplemental Figure 3E). Each node of a *vks1* plant was shorter than the wild type. Fewer and shorter aerial and lateral roots were produced by *vks1* than by the wild type (Supplemental Figure 3F). Furthermore, the segregation ratio of mutant and wild-type seeds from self-pollinated heterozygous plants (*vks1*/*Vks1*) was significantly lower than the expected Mendelian ratio of 1:3 (Supplemental Figures 4A and 4B), although seed set in crosses A619 × *vks1* and *vks1* × A619 was normal (Figures 1C and 1D). Again, *vks1* seeds displayed dramatically greater kernel weight variation compared with wild-type seeds from the same segregating ear (Supplemental Figure 4C). We inspected *vks1* pollen with a light microscope and found that ∼30% was infertile (Supplemental Figures 4D to 4F). Therefore, the *vks1* mutation appears to have pleiotropic effects on plant development.

### *Vks1* Encodes a Kinesin-14 Motor Protein

**Figure 2.**
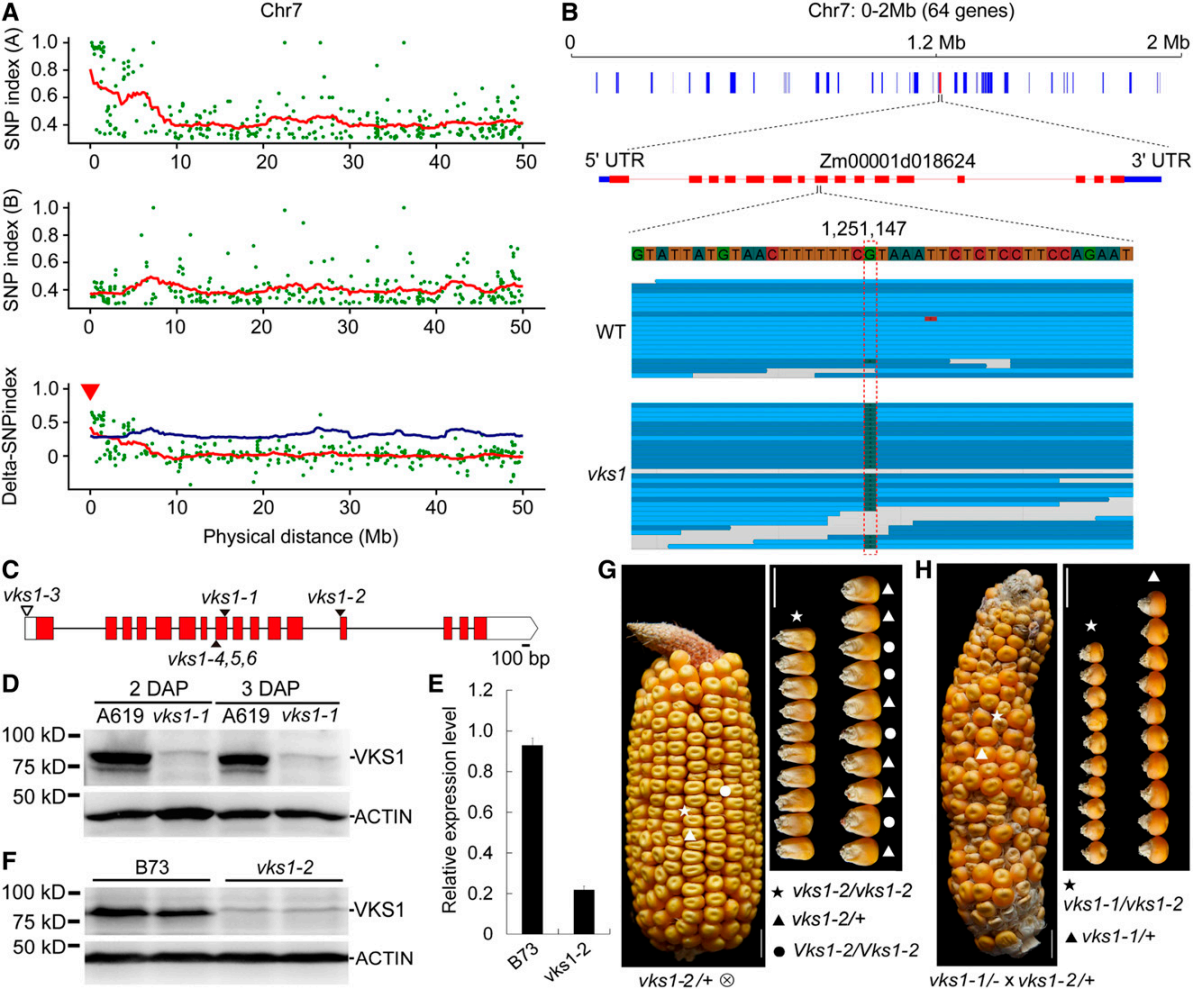
Map-Based Cloning and Genetic Confirmation of *vks1*. **(A)** Mapping by sequencing of *vks1*. Ratios of SNP index (A619/−) between different pools of segregating phenotypic classes (top panel, normal phenotype in pool A: A619/A619 and A619/−; middle panel, *vks1* phenotype in pool B: −/−; and bottom panel, SNP index between the top and middle panels) are shown for a region near the left terminal end of chromosome 7. **(B)** The *vks1* mapping interval. An SNP in the eighth exon of Zm00001d018624 results in a CGA-to-TGA transition in *vks1* mutants. UTR, untranslated region. **(C)** Schematic representation of *Vks1* gene structure with the mutant alleles indicated. Red rectangles and black lines indicate exons and introns, respectively. The black triangles indicate the mutation sites in *vks1-1*, *vks1-2*, and *vks1-4,5,6* alleles, and the white triangle indicates the *Mu* insertion in the *vks1-3* allele. **(D)** Immunoblotting analysis of VKS1 in seeds of A619 and *vks1-1*. ACTIN was used as an internal control. The sizes of proteins are indicated beside the gels. **(E)** Expression levels of *Vks1* in seeds of B73 and *vks1-2* at 2 DAP. **(F)** Immunoblotting analysis of VKS1 in seeds of B73 and *vks1-2* at 2 DAP. ACTIN was used as an internal control. The sizes of proteins are indicated beside the gels. **(G)** The kernel phenotype of *vks1-2*. Left panel, a self-pollinated *vks1-2*/+ ear segregating *vks1* (as indicated by an asterisk) and normal (as indicated by a dot and a triangle) seeds. Right panel, all small kernels were determined to be homozygous for *vks1-2*, while normal kernels bore one (as indicated by triangles) or two wild-type (as indicated by dots) alleles. Bar = 1 cm **(H)** Allelic test of *vks1-1* and *vks1-2* alleles. Left panel, a representative ear from a homozygous *vks1-1* plant pollinated by *vks1-2/+* pollen segregating *vks1* and normal seeds. Right panel, all small kernels were determined to be the genotype *vks1-1*/*vks1-2*, while all normal seeds were *vks1-1*/+. Bar = 1 cm.

To map the mutant gene, plants of *vks1* and its wild type (A619) were crossed and then self-pollinated. The resulting ears segregated mutant and normal seeds (Supplemental Figure 4A). More than 100 seeds of each phenotype were germinated for DNA extraction. We performed whole-genome sequencing of the two DNA pools. Comparison of the single-nucleotide polymorphism (SNP) index between the *vks1* and wild-type pools revealed a region near the left terminal end of chromosome 7, which contains 64 genes (Figure 2A). Genomic sequencing of *vks1* mutants revealed an SNP in the eighth exon of Zm00001d018624, which resulted in a CGA-to-TGA transition, leading to a premature stop codon in its coding sequence (Figures 2B and 2C) and therefore a dramatic reduction in its transcript level (Figure 2D). Subsequent immunoblotting analysis of VKS1 revealed that this protein was absent in developing *vks1* seeds (Figure 2E), indicating that *vks1* is a null mutant for this gene. We also used *vks1* to create a BC1F1 population with the B73 inbred line for traditional map-based cloning, by which *vks1* was narrowed down to an interval between the markers InDel8450 and InDel5839 (Supplemental Figures 5A and 5B). Based on the B73 genome, these two markers span ∼130 kb, where only seven genes were annotated (Supplemental Figure 5B). The *vks1* phenotype was completely linked to the homozygous TGA, whereas all normal seeds were heterozygous at this position (Supplemental Figure 5C). These results confirmed that the disrupted Zm00001d018624 was associated with the mutant phenotype.

To validate that this mutation was responsible for the *vks1* phenotype, we performed a reverse genetic screen for additional alleles in the Maize EMS-induced Mutant Database (http://www.elabcaas.cn/memd/) and the Maize Genetics Cooperation Stock Center (https://www.maizegdb.org/uniformmu). This screen resulted in the identification of two alleles of *vks1*, one each from the Maize EMS-induced Mutant Database (mutant ID: EMS4-092f0c) and the Maize Genetics Cooperation Stock Center (mutant ID: UFMu-01867). The cloned *vks1* allele was designated *vks1-1*, and the additional alleles were designated *vks1-2* (with an EMS-induced mutation in the B73 background) and *vks1-3* (with the *Mu* transposable element insertion in the W22 background; Figure 2C). The *vks1-2* allele is caused by a mutation at the splice acceptor site in the 12th intron of Zm00001d018624, which results in defective RNA splicing of this intron. The *vks1-3* allele results from a *Mu* insertion in the 5′ untranslated region (UTR) of Zm00001d018624 (Figure 2C; Supplemental Figures 5D and 5E). Self-pollinated *vks1-2/+* ears segregated small seeds of varying sizes, as observed in *vks1-1* (Figures 1B and 2F; Supplemental Figure 4A). *vks1-3* seeds were also much smaller than their wild-type counterparts (W22) but did not display conspicuous seed size variation on homozygous *vks1-3* ears (Supplemental Figure 5D), suggesting that the *vks1* phenotype was partially genetic background dependent. To perform the allelism test, we made a cross between homozygous *vks1-1* and heterozygous *vks1-2*/+ plants. The resulting ears segregated *vks1* and wild-type seeds at a ratio of nearly 1:1 (Figure 2G).

In addition, we used the clustered regularly interspaced short palindromic repeats (CRISPR)/ CRISPR associated protein 9 (Cas9) technology to create more *vks1* null alleles. The gRNA was designed to target the eighth exon of Zm00001d018624 (Supplemental Figure 5F). Three independent transgenic lines, which bear a C deletion, a T insertion, and a 127-bp deletion, respectively, were obtained and designated *vks1-4*, *vks1-5*, and *vks1-6* (Figure 2C; Supplemental Figure 5F). Self-pollinated *vks1-4/+* ears (as a representative for these CRISPR/Cas9-induced null mutants) also segregated small seeds of varying sizes (Supplemental Figure 5G), as observed in *vks1-1* and *vks1-2* (Figures 1B and 2F). Therefore, Zm00001d018624 is the gene responsible for the *vks1* phenotype.

**Figure 3.**
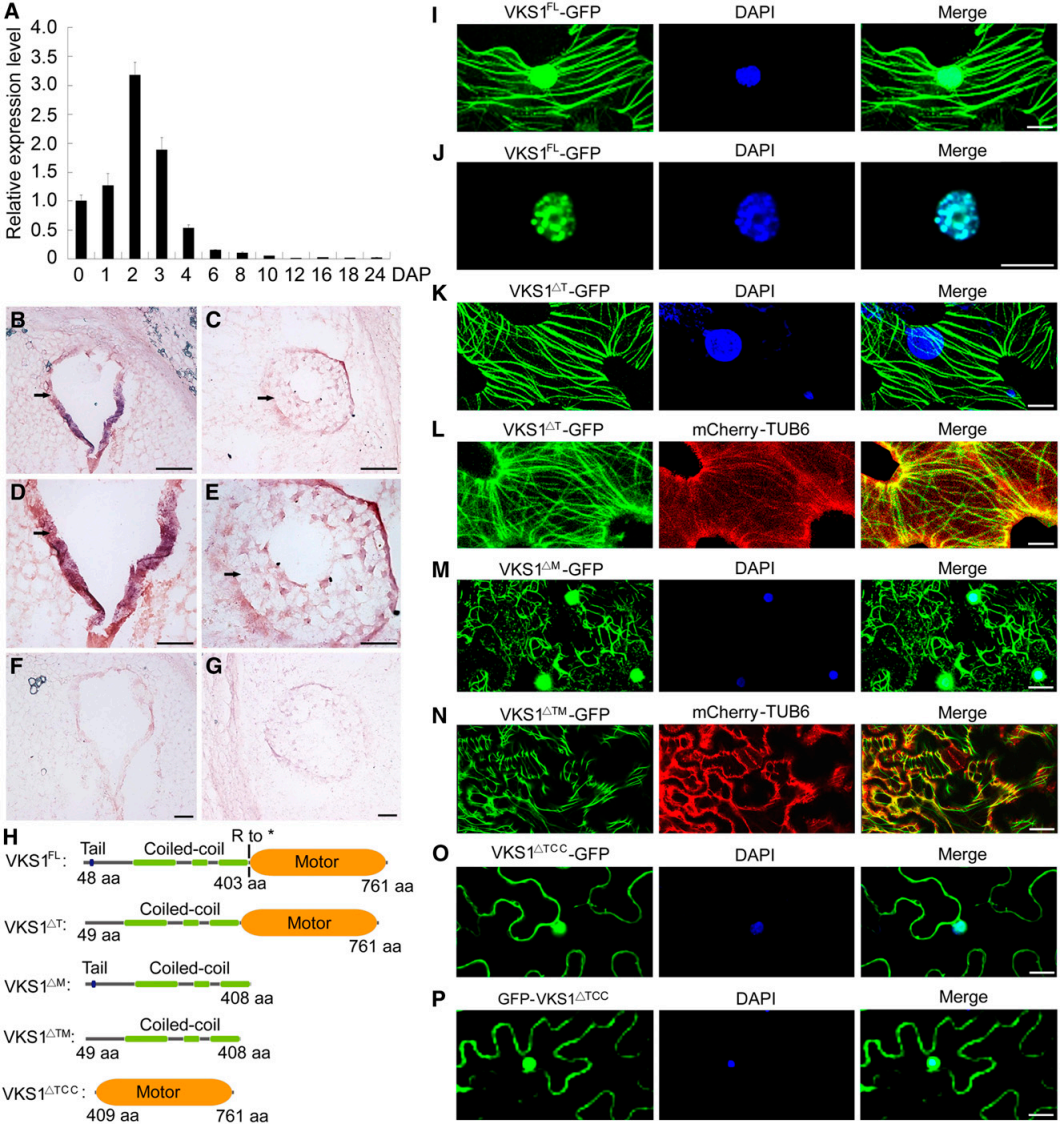
The Gene Expression Pattern of *VKS1* and Its Protein Subcellular Localization. **(A)** RT-qPCR analysis of *Vks1* during seed development. All expression levels are normalized to *Actin*. The expression level of *Vks1* at 0 DAP is set to 1. Error bars represent sd from three biological replicates. **(B)** to **(G)** RNA in situ hybridization of *Vks1* in seeds at 2 and 3 DAP. Positive signals (shown in red) are clearly restricted to the coenocyte at 2 DAP (**[B]** and **[D]**) and endosperm cells engaging in cellularization at 3 DAP (**[C]** and **[E]**). When hybridized with sense probes, no signal is observed in the sections of seeds at 2 DAP **(F)** and 3 DAP **(G)**. Bars = 50 μm. **(H)** Schematic diagrams of constructs for subcellular localization. VKS1^FL^, VKS1^△T^, VKS1^△M^, VKS1^△TM^, and VKS1^△TCC^ represent the full-length VKS1 protein and fragments lacking the tail, the motor, both the tail and motor domains, and both the tail and CC domains, respectively. aa, amino acids. **(I)** Subcellular localization of VKS1^FL^-GFP. The nucleus is stained with DAPI. **(J)** Localization of VKS1^FL^-GFP in the nucleus. The nucleus is stained with DAPI. **(K)** Subcellular localization of VKS1^△T^-GFP. The nucleus is stained with DAPI. **(L)** Colocalization of VKS1^△T^-GFP and mCherry-TUB6. **(M)** Subcellular localization of VKS1^△M^-GFP. The nucleus is stained with DAPI. **(N)** Colocalization of VKS1^△TM^-GFP and mCherry-TUB6. **(O)** and **(P)** Subcellular localization of VKS1^△TCC^-GFP and GFP-VKS1^△TCC^. The nucleus is stained with DAPI. For **(I)** to **(P)**, bars = 10 μm.

*Vks1* consists of 16 exons and 15 introns (Figure 2C), and it encodes a kinesin-14A protein (ZmKIN11) with 761 amino acids (Lawrence et al., 2002). Protein families database (Pfam) searches (http://pfam.xfam.org/search) indicated that this protein contains a tail domain, a coiled-coil (CC) domain, and the classical C-terminal motor domain (Figure 3H). A total of 59 kinesin proteins were found in the maize B73 genome (RefGen_v4) and were grouped into 10 subfamilies based on the kinesin sequences from Arabidopsis and rice (Supplemental Figure 1; Supplemental File).

VKS1 shares 94% similarity with KINDR, which is encoded by Ab10 and is involved in the activation of neocentromeres that promote meiotic drive in maize. *Kindr* is a cluster of at least eight kinesin genes, and all the encoded proteins possess only a partial CC domain that is present as an intact domain in VKS1 (Dawe et al., 2018). In addition, VKS1 has another homolog in maize, the classical plant kinesin DV1, which is required for meiotic spindle pole organization (Higgins et al., 2016). In the grass family, 21 VKS1 homologs with a high similarity (>60%) were characterized from nine species in the public database (Supplemental Figure 6), suggesting a conserved function for this gene.

### *Vks1* Is Predominantly Expressed during Early Endosperm Development

To infer the function of VKS1 in maize endosperm development, we examined its temporal and spatial expression patterns. Since it is difficult to cleanly dissect endosperm from maternal tissues at early developmental stages, whole seeds from 0 to 8 DAP were used for RNA extraction. At later stages, the endosperm could be easily separated from the pericarp and embryo. RT-qPCR revealed that *Vks1* is predominantly expressed in early seed development. The expression of *Vks1* increased immediately after double fertilization and reached a climax at 2 DAP. Thereafter, the level of *Vks1* transcripts was sharply reduced. At 8 DAP, only basal expression was detected (Figure 3A). The temporal expression pattern corresponded well with the period of early endosperm development.

To examine its spatial expression, we performed RNA in situ hybridization with a *Vks1* antisense probe at 2 and 3 DAP, the time points when *Vks1* transcripts are most highly abundant. This revealed that *Vks1* is expressed in the coenocyte at 2 DAP (Figures 3B and 3D). At 3 DAP, the endosperm was undergoing rapid cellularization. The repetition of mitotic divisions four or five times results in a complete cellular endosperm by 4 DAP in maize (Olsen, 2004). Although the strongest hybridization signal was still present in the outermost endosperm cell layer, it expanded to the entire cellularizing endosperm (Figures 3C and 3E). *Vks1* was not detected in maternal tissues (pericarp and nucellus), and it appears that *Vks1* is endosperm-specific during early seed development. In control experiments, the *Vks1* sense probe failed to produce a visible signal (Figures 3F and 3G).

### Functions of Different VKS1 Domains in Subcellular Localization

To determine the subcellular localization of VKS1 and the function of its different domains, the full-length protein (VKS1^FL^, amino acids 1–761) and fragments lacking the tail (VKS1^△T^, amino acids 49–761), the motor (VKS1^△M^, amino acids 1–408), the tail and motor domains (VKS1^△TM^, amino acids 49–408), or the tail and CC domains (VKS1^△TCC^, amino acids 409–761) were fused to the N terminus of green fluorescent protein (GFP; Figure 3H). We also generated a construct (GFP-VKS1^△TCC^) in which the motor domain was ligated to the C terminus of the GFP. The β-tubulin6 protein was fused to mCherry. The resulting constructs driven by the constitutive 35S promoter were transiently expressed in *Nicotiana benthamiana* leaves. VKS1^FL^-GFP containing the entire VKS1 protein localized to cortical MTs and to the nucleus (Figure 3I). At higher magnification, VKS1^FL^-GFP signals appeared to be merged precisely with chromatin in the nucleus (Figure 3J). VKS1^△T^-GFP lacking the tail domain failed to enter the nucleus but was still able to localize to MTs, indicating that the tail domain harbors a signal for nuclear localization (Figures 3K and 3L). Without the motor domain in VKS1^△M^-GFP, localization to the nucleus and MTs was not affected, but the architecture of MTs was disrupted and displayed a morphologically disordered assembly in the cell, indicating that the motor domain was critical for correct protein-protein interaction with MTs (Figure 3M). Similar to VKS1^△M^-GFP, VKS1^△TM^-GFP exhibited severely distorted MT structure and failed to locate in the nucleus (Figure 3N). The VKS1^△TCC^-GFP and GFP-VKS1^△TCC^ constructs possessing only the motor domain were both ubiquitously distributed in the nucleus and cytoplasm, indicating that the CC domain was essential for MT localization (Figures 3O and 3P).

### Defects in Nuclei Migration in the Coenocyte

**Figure 4.**
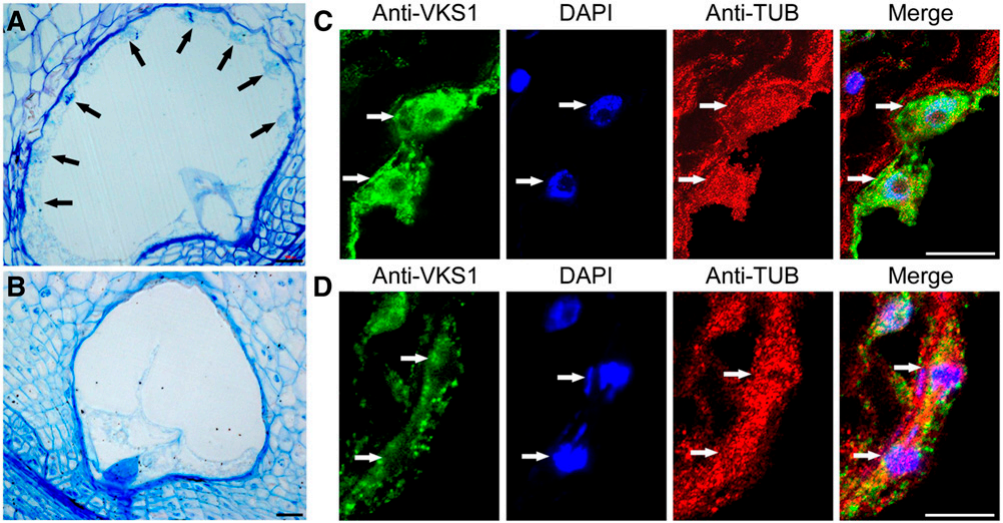
Phenotypes of the Coenocyte in the Wild Type and *vks1*. **(A)** and **(B)** Semithin sections of A619 and *vks1* seeds at the coenocytic stage (2 DAP). Arrows indicate the free nuclei evenly distributed along the peripheral region of the cell in the A619 wild type **(A)**, while nuclei are not visible in *vks1* **(B)**. Bars = 50 μm. **(C)** and **(D)** Colocalization of VKS1 and MTs in the coenocyte as shown by immunofluorescence assays in the A619 wild type **(C)** and *vks1* **(D)**. Bars = 20 μm.

Since *Vks1* is most highly expressed in the coenocyte, semithin sections of 2-DAP-old endosperms of the wild type and *vks1* were investigated by light microscopy. At this stage, wild-type endosperm nuclei had experienced multiple rounds of mitosis without cytokinesis, forming a multinucleate coenocyte. As shown in Figure 4A, the nuclei migrated and evenly occupied positions along the peripheral cell wall layer, sharing a common cytoplasm and a large central vacuole. We performed immunohistochemical analysis with antibodies raised against VKS1 and α-tubulin, which were in turn recognized by secondary antibodies labeled with GFP and red fluorescent protein, respectively. This assay demonstrated that each free nucleus was surrounded by an interactive MT system that colocalized with the VKS1 protein and formed relatively discrete territorial domains (Figure 4C). In contrast, nuclei in the *vks1* coenocyte could not be clearly seen (Figure 4B). Since *vks1-1* is a null mutant that does not accumulate the VKS1 protein (Figure 2E), the residual GFP signal probably resulted from background noise or cross-reaction with homologs. In the absence of VKS1, the MT system surrounding the nucleus exhibited an abnormal morphology. The boundary between adjacent MT domains was not formed. More seriously, the nuclei in the *vks1* mutant appeared to have defects in migration. They seemed to adhere more closely to the coenocytic cell membrane (Figure 4D), which made them unrecognizable without the aid of 4′,6-diamidino-2-phenylindole (DAPI) staining at lower resolution (Figure 4B). Dispersed chromosomes were observed in the vicinity of the nuclei (Figure 4D). These results suggest that VKS1 is essential for the establishment of MT systems for nuclei in the coenocyte.

### VKS1 Colocalizes with MTs during Early Mitotic Divisions

Similar to the coenocytic stage, cellularization also occurs rapidly. By 3 to 4 DAP, the endosperm is fully cellularized (Figures 1G and 1H), which creates difficulties tracking the process of nuclear and cellular division. To understand the function of VKS1 in mitosis and cytokinesis, we investigated endosperm cells at 6 DAP, when early mitotic divisions were common.

To examine dynamic localizations of VKS1 during mitotic division, we made a series of paraffin sections of A619 endosperms and performed immunohistochemical analysis of VKS1 and α-tubulin proteins (Supplemental Figure 7). Mitosis is divided into multiple phases: prophase, metaphase, anaphase, and telophase. In interphase, VKS1, α-tubulin, and the loosely packed chromatin colocalized in the nucleus, where the three signals seemed to overlap seamlessly (Supplemental Figure 7A). In prophase, as chromatin fibers condensed into discrete chromosomes, the VKS1 protein maintained colocalization with α-tubulin at all times (Supplemental Figure 7B), suggesting that VKS1 is involved in the initiation of mitotic spindle formation. In metaphase, the MTs were attached to the kinetochores of sister chromatids that lined up neatly along the equator of the cell, and the VKS1 protein was distributed on the spindle fibers (Supplemental Figure 7C). In late anaphase and early telophase, as sister chromatids pulled apart to the opposite poles, the spindle began to break down (Supplemental Figure 7D). VKS1 and α-tubulin appeared to relocate together to the central region of the cell, where the phragmoplast was assembled, suggesting that VKS1 is required for MT-mediated phragmoplast formation.

**Figure 5.**
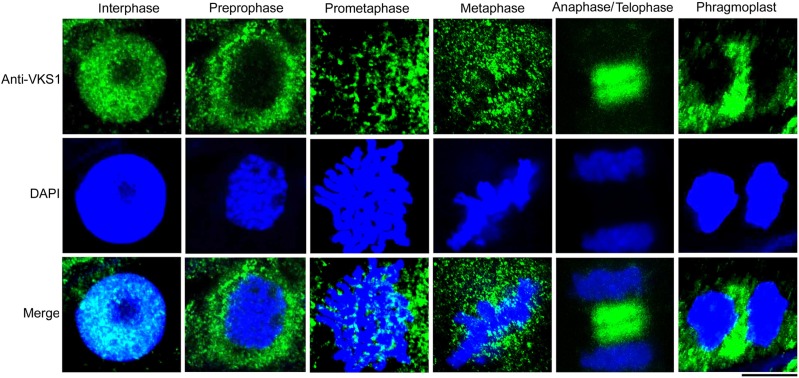
Dynamic Localization of VKS1 during Mitosis. Localization of the VKS1 protein in early endosperm cells during mitosis was assayed by immunofluorescence. VKS1 signaling is colored green, and the nucleus is colored blue. Bar = 10 μm.

Since paraffin embedding and processing can cause morphological alterations that may not reflect the original in vivo structures, we performed immunohistochemical examinations of endosperm cells without embedding and dehydration (see Methods). Consistent with the observation in Supplemental Figure 7, the localizations of VKS1 were dynamic, following the process of mitotic division. In interphase, VKS1 colocalized with the chromatin surrounded by the nuclear envelope. During mitosis, VKS1 was relocated in a dynamic pattern by which the spindle was assembled and the phragmoplast was formed (Figure 5). Specifically, immunohistochemical assays clearly showed that VKS1 colocalized with MTs during the initiation of phragmoplast formation (Supplemental Figure 8).

### Defects in Mitosis and Cytokinesis in the *vks1* Mutant

**Figure 6.**
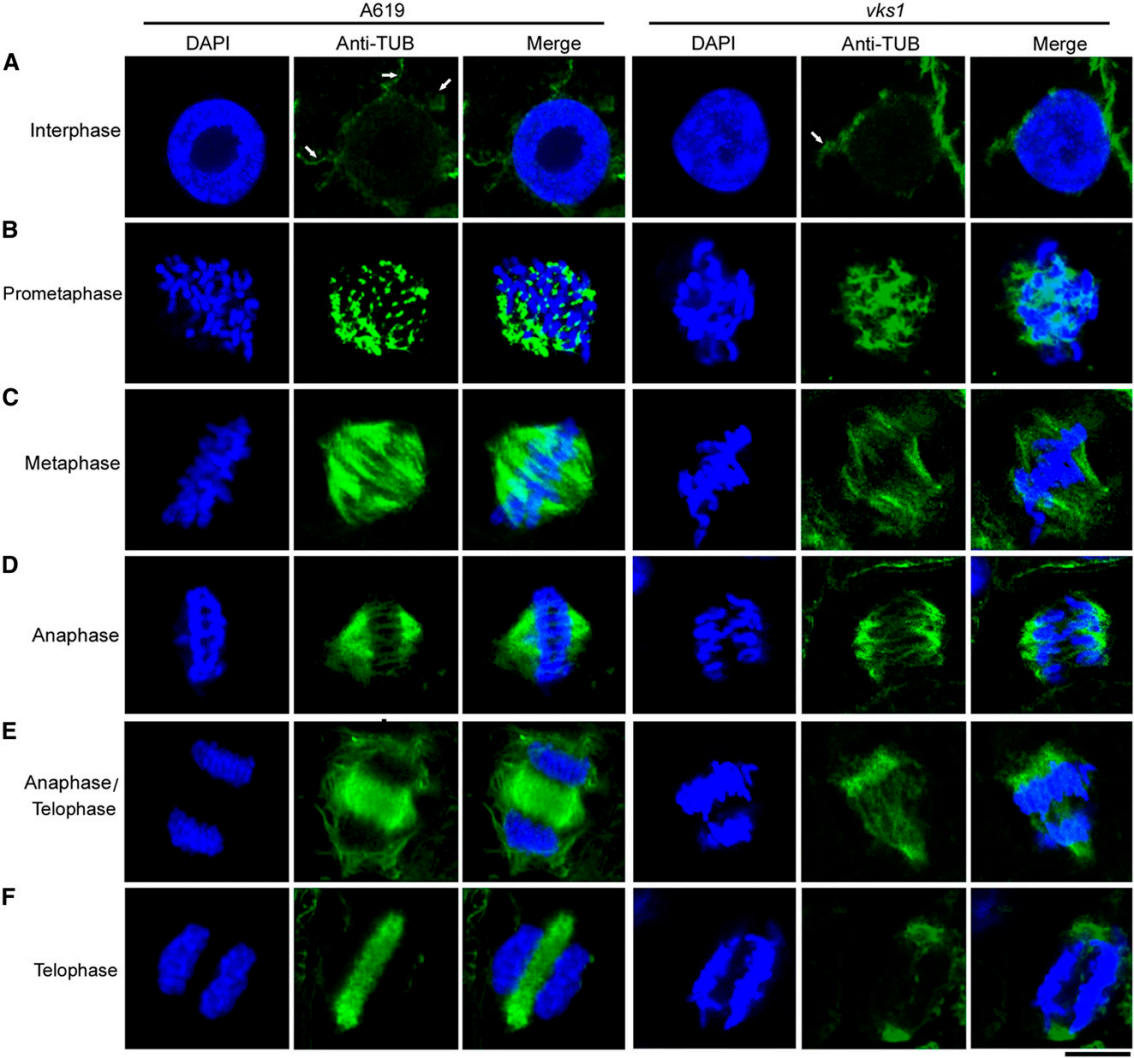
Early Endosperm Mitotic Divisions in the Wild Type and *vks1*. MT arrays were visualized by immunostaining with anti-α-tubulin during mitosis in wild-type and *vks1* endosperm cells at 6 DAP. MTs are colored green, and nuclei or chromosomes are colored blue. Phases are as follows: interphase **(A)**; prometaphase **(B)**; metaphase **(C)**; anaphase **(D)**; anaphase/telophase **(E)**; and telophase **(F)**. Bar = 10 μm.

To further investigate the role of VKS1 in each phase of mitosis in early endosperm development, we performed immunostaining of α-tubulin to visualize MT arrays in *vks1* and the wild type. In interphase, MTs were observed to be nucleated from the surface of the nuclear envelope and radiated into the cell, forming an array of fibers in the wild type (Figure 6A). As higher plants do not possess centrosomes, this structure is thought to function as the microtubule-organizing center (MTOC). There were fewer radial MTs in *vks1* than in the wild type (Figure 6A). In premetaphase, because the cells of higher plants (such as maize) lack centrosomes, MTs are organized into a spindle by chromosomes themselves. Although the chromatin in *vks1* was condensed into thread-like chromosomes, as observed in the wild type, the shapes of MTs were remarkably different. The formation of a spindle was already initiated in the wild type, wherein the MTs were stretched to attach to the chromosomes. In contrast, the MTs in *vks1* exhibited an amorphous arrangement, and their attachment to the chromosomes appeared as a mass of tangles (Figure 6B). In metaphase, spindle fibers in the wild type were concentrated to a tip on each opposite pole, forming an umbrella-like shape. The chromosomes were aligned along the center of the cell; in contrast, the *vks1* cell formed a spindle with splayed poles and looser and fewer fibers. The chromosomes appeared to be misarranged. In addition, some sister chromatids were not correctly attached to spindle fibers (Figure 6C; Supplemental Figure 9A).

When anaphase was reached, the umbrella-shaped spindle in the wild type began to pull the chromosomes toward opposite sides of the cell, whereas the flattened-pole spindle in *vks1* had apparent defects in moving the chromosomes. Sister chromatids were not evenly attached to the irregular spindle fibers on each side and moved in opposite directions in a skewed rather than a straight line (Figure 6D; Supplemental Figure 9B). In late anaphase and early telophase in the wild type, the daughter chromosomes were equally pulled to the opposite ends of the cell. The spindle began to disappear, and the MTs began to accumulate in the middle of two daughter nuclei to initiate phragmoplast formation (Figure 6E); however, in *vks1*, the chromosomes were not equally allocated to each side of the cell (Figure 6E). The cohesins that bind sister chromatids together might not have been completely cleaved in anaphase (Supplemental Figure 9B), leading some sister chromosomes to be pulled to one side of the cell. Later in wild-type telophase, MTs continued to be enriched in the phragmoplast and finally developed a long and dense band across the entire cell center in the wild type. In contrast, in *vks1*, fewer MTs were deposited in the region where the phragmoplast was expected to be assembled (Figures 6E and 6F; Supplemental Figures 9C and 9D). Overall, these results demonstrate that VKS1 is critical for mitotic divisions in early endosperm development.

**Figure 7.**
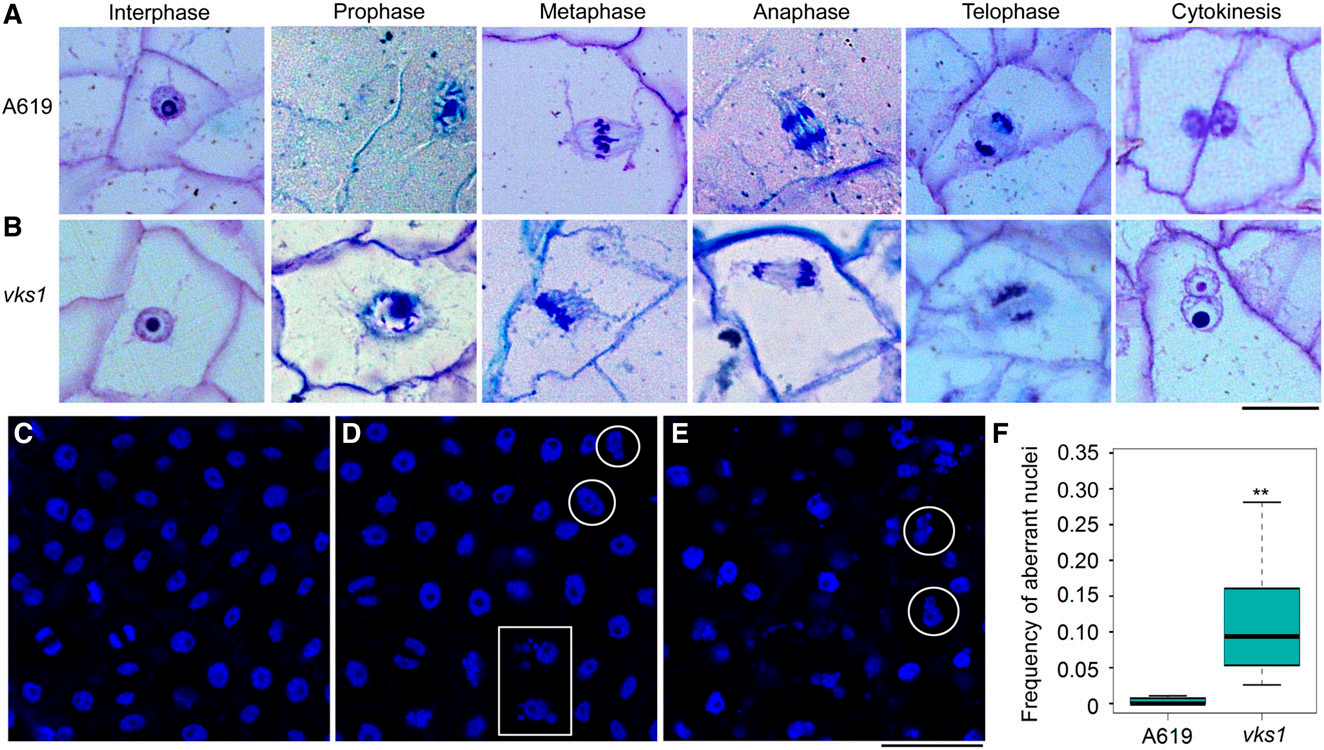
The Phenotypes of Endosperm Cells and Nuclei in A619 and *vks1* during Early Development. **(A)** and **(B)** Endosperm cells undergoing mitosis and cytokinesis in A619 **(A)** and *vks1* **(B)** at 6 DAP. Bar = 25 μm. **(C)** to **(E)** Nuclei of endosperm cells during mitosis in A619 **(C)** and *vks1* (**[D]** and **[E]**) at 6 DAP. **(D)** shows a mild phenotype and **(E)** shows a severe phenotype of *vks1*. The nuclei stained by DAPI are in blue. Bar = 50 μm. **(F)** Frequency of aberrant nuclei in endosperms of A619 and *vks1* at 6 DAP. More than 4000 nuclei of A619 and 2600 nuclei of *vks1* from 20 microscope fields were observed. The mean value of the ratios of aberrant nuclei to total nuclei in each field of A619 or *vks1* was used to represent the frequency of aberrant nuclei in endosperm. **, P < 0.01 as determined by Student’s *t* test.

The absence of VKS1 resulted in abnormal mitosis in *vks1* (Figure 6), which may in turn have affected cell division in the endosperm. To examine this possibility, semithin sections of endosperms at 6 DAP were inspected. Endosperm cells at different stages that were characteristic of normal mitosis and cytokinesis were observed in the wild type (Figure 7A). In telophase, the phragmoplast between two sets of separated daughter chromosomes was transversely elongated to the edges of the cell. During cytokinesis, the cell wall developed based on the phragmoplast divided the cell into two daughter cells. In *vks1*, defects in mitosis were consistent with the observation presented in Figure 6. Because of the malformed spindle and incorrectly aligned sister chromatids of metaphase, the daughter chromosomes were unequally separated. The defective phragmoplast resulted in the failure of cell division; thus, the affected *vks1* cell contained one large nucleus and one small nucleus (Figure 7B).

To illustrate the substantial deformities in *vks1* in larger view, a field of nuclei stained by DAPI was examined. Compared with normal nuclei and chromosomes in the wild type (Figure 7C), aberrant nuclei were frequently observed in *vks1* endosperm cells. A mild phenotype is shown in Figure 7D, and a severe phenotype is shown in Figure 7E. The conjugate nuclei of different sizes indicated by the circles likely resulted from unsuccessful cytokinesis (Figures 7D and 7E). The micronuclei (indicated by the box) probably resulted from unequal chromosome separations (Figure 7E). When calculating the frequency of abnormal nuclei in the wild type and *vks1*, the nuclei in wild-type endosperm cells were almost normal, whereas the ratios of abnormal nuclei in *vks1* ranged from 2.5 to 34.4% (Figure 7F). Taken together, these results demonstrate that VKS1 is essential for mitosis and cytokinesis in early endosperm development.

## DISCUSSION

### Mutation in *vks1* Affects Early Maize Endosperm Development

Yield is determined by many factors, including seed size. In maize kernels, the endosperm accounts for 90% of seed dry mass and is the major organ for nutrient storage, thereby predominantly determining the final size of the seed. Cell number and cell size are both important for endosperm volume (Sabelli and Larkins, 2009a). Although considerable progress has been made understanding endosperm cell expansion associated with storage reserve synthesis and accumulation, far less is known about the genes that regulate cell number during early endosperm development. Endosperm formation begins after double fertilization, and it initially develops as a coenocyte in which the nucleus undergoes multiple rounds of division without cytokinesis, forming a multinucleate cell. Cellularization initiates 2 to 3 DAP and is usually completed by 4 DAP, which is followed by a period of frequent early mitotic divisions (Sabelli and Larkins, 2009a). In Arabidopsis, mutations in genes encoding the VQ motif protein HAIKU1, the Leucine-rich repeat receptor IKU2, and the WRKY transcription factor MINISEED3 resulted in precocious cellularization of the endosperm, thereby leading to reduced seed size (Luo et al., 2005; Zhou et al., 2009; Wang et al., 2010). This indicates that the timing of the transition from the coenocytic stage to cellularization stage is a key decision point in endosperm development. Early mitotic divisions are another critical step, as most cells of the mature endosperm are generated during this phase (Sabelli et al., 2013, 2018). Although early endosperm development directly contributes little to kernel biomass, the cell number and endosperm structure established during this stage create the demand for subsequent accumulation of storage reserves.

In maize, while numerous mutants show seed defects, few have been reported where the phenotype specifically features aberrant early endosperm development. Indeed, an abundance of mutants with reduced kernel size and weight have been cloned and characterized, but most of them have been found to result from defective synthesis and accumulation of storage reserves and thus often display a phenotype that is characteristic of a certain mutation of endosperm development (e.g., the *opaque*, *floury*, and *shrunken* mutants). In general, for maize genetics, a major strategy for identifying and characterizing genes involved in a pathway is mutational inactivation. However, screening mutations that specifically affect early endosperm development is challenging because mutants of this kind often have pleiotropic effects on seed or plant development, with an aborted, small, or even nonviable phenotype, such as in *globby1*, *empty pericarp*, and *defective kernel* (*dek*) mutants (Sheridan and Neuffer, 1980; Scanlon et al., 1994; Costa et al., 2003). In early efforts to investigate maize seed development, a group of *dek* mutants were described. These mutants generally have decreased mitotic activity, thereby resulting in greatly reduced cell numbers; however, none of these mutants are viable (Kowles et al., 1992). Therefore, these mutants are not specifically involved in early endosperm development.

The *vks1* mutant was identified through a screen of an EMS-induced mutant collection. This mutant drew our attention because its kernel size not only was small but also varied dramatically among different progeny (Figures 1A and 1B; Supplemental Figure 2A). By recurrent backcrossing, this phenotypic variation was confirmed to result from a single-gene mutation rather than the combined effects of multiple mutant loci. The single-kernel weight of the wild type (A619) showed limited variation, whereas in *vks1* it ranged from 0.0128 to 0.1898 g. Examination of endosperm development at different stages demonstrated that the small kernels of *vks1* were mainly caused by a reduction in cell number rather than a reduction in cell size. A reduction in cell number in *vks1* was observed as early as 4 DAP (Figures 1G to 1N), but grain filling appeared to be normal in late endosperm development (Supplemental Figures 3A and 3B). At maturity, the storage proteins and starch contents showed no perceivable difference from those in the wild type (Supplemental Figures 3C and 3D), indicating that VKS1 primarily functions at the early phases of endosperm development.

More importantly, despite substantial variation in kernel size, the germination of *vks1* seeds was comparable to that of wild-type seeds, indicating that embryo development was apparently not impaired by the *vks1* mutation. The *vks1* plants were shorter than the wild-type ones, but their vegetative growth exhibited no conspicuous abnormalities. The homozygous ears and the ears from reciprocal crosses between the wild type and *vks1* showed normal seed set. However, the segregation ratio of *vks1* and normal seeds in self-pollinated heterozygous ears deviated from that expected under Mendelian inheritance. This discrepancy was likely caused by the viability of *vks1* pollen (Supplemental Figures 4D to 4F), which was lower than the viability of pollen bearing the wild-type *Vks1* allele in terms of completing pollination.

Overall, the *vks1* mutant displays a previously undescribed phenotype in maize endosperm development. Its effects are mainly restricted to early stages, although it also has some adverse effects on plant development. Cloning and characterization of this mutant gene are valuable for understanding the impact of early maize endosperm development.

### VKS1, a Kinesin-14 Protein, Dually Localized to MTs and the Nucleus

*Vks1* was determined to encode ZmKIN11, which belongs to the kinesin-14A subfamily (Lawrence et al., 2002; Dawe et al., 2018). VKS1 has two homologs in the maize genome, KINDR and DV1 (ZmKin6). KINDR is encoded by a gene cluster that is present only on Ab10. KINDR converts heterochromatic regions called knobs into neocentromeres, thereby promoting meiotic drive in maize. Epimutation of the *Kindr* cluster induced by RNA interference results in the loss of meiotic drive but no apparent defects in endosperm development (Dawe et al., 2018). DV1, encoded by Zm00001d002186 on chromosome 2, is required for meiotic spindle pole organization. Mutation of *dv1* causes failure in promoting the formation of finely focused spindle poles during meiosis, thereby leading to aberrant chromosome segregation and a higher pollen abortion rate, but plants carrying this mutation are phenotypically indistinguishable from their wild-type siblings, indicating that *dv1* does not cause deleterious effects on mitosis (Staiger and Cande, 1990). Similar to mutation of *dv1*, mutation of *vks1* causes pollen abortion at a certain rate (Supplemental Figure 4E), indicating that VKS1 and DV1 are functionally redundant in pollen meiosis but may be greatly diverged in mitosis.

This raises the following question: is the divergence of coding sequences or the regulation of expression of *Vks1* and *dv1* responsible for the functional divergence? Although *Vks1* is predominantly expressed in early stages of endosperm development, it is also expressed in shoots, leaves, tassels, and shoot apical meristems where mitosis and cytokinesis actively occur for cell proliferation based on the public RNA sequencing data (Chen et al., 2014). Interestingly, the temporal expression patterns of *Vks1* and *dv1* during endosperm development are similar (Supplemental Figure 10), although whether they have a spatial expression overlap is unknown. Since VKS1 and DV1 share >60% similarity, we do not exclude the possibility that VKS1 and DV1 also have partial functional redundancy in mitosis. It is intriguing to create a double mutant of these two genes to test this possibility. Mitosis and cytokinesis are fundamental mechanisms for cell proliferation. Mutations in *vks1* only cause a small and variable seed size rather than the *dek* phenotype, and not all nuclei in the *vks1* endosperm are abnormal (Figure 7F), indicating that other kinesin-14 proteins may have functional redundancy with VKS1. Indeed, *Kin4A* (*Zm00001d019670*), *Kin3* (*Zm00001d010790*), and *Kin14C* (*Zm00001d043504*) in the kinesin-14 subfamily are also highly expressed at early endosperm stages (Supplemental Figure 10; Chen et al., 2014), implying that they have an important role in early endosperm development. Because the basic processes of early endosperm development are similar in grasses, we speculate that the functions of VKS1 homologs in other species (Supplemental Figure 6) are conserved in regulating mitosis and cytokinesis during early endosperm development.

VKS1 is predicted to contain three domains (the tail, CC, and motor domains; Figure 3H), each having a specific function for subcellular localization. The full-length VKS1 protein localizes to MTs and the nucleus (Figures 3I and 3J). The tail contains a signal for nuclear localization (Figures 3J and 3K), the CC domain is required for MT localization (Figure 3O), and the motor is essential for its correct localization to MTs (Figures 3M and 3N). Our data are consistent with the observation in which KINDR, the homolog of VKS1 bearing a deletion in the CC domain, localizes specifically to chromosome knobs rather than to the MTs (Dawe et al., 2018). Based on variations in protein structure and subcellular localization, this indicates that the functions of VKS1 and KINDR proteins have diverged.

### VKS1 Is Essential for Nuclear-Cytoplasmic Domain Formation in the Coenocyte

*Vks1* transcription occurred throughout early endosperm development, with a peak at 2 DAP, but it was not detected in the mid and late phases (Figure 3A). Examination of the spatial distribution of *VKS1* mRNAs in 2- and 3-DAP-old developing seeds showed that the expression of *Vks1* was restricted to the coenocyte and cellularizing endosperm cells (Figures 3B to 3E), consistent with the observation that the cell number reduction in *vks1* occurred in the early phase of endosperm development. After double fertilization, the primary endosperm nucleus undergoes several rounds of nuclear division without cell wall formation and cytokinesis. These divisions appear to be synchronous within day 1 after double fertilization, but later, only the neighboring nuclei divide synchronously. This is likely due to developmental gradients formed from the embryo sac to the chalazal region (Sabelli and Larkins, 2009b). In maize, as many as 512 nuclei can be generated during coenocytic nuclear proliferation (Leroux et al., 2014). We did not determine whether the number of coenocytic nuclei in *vks1* was reduced, but we observed abnormal nuclei division and distribution in the coenocyte (Figure 4), which may result in a slow start for the rest of kernel development and consequently a small kernel size. At 2 DAP, the nuclei were evenly spaced at the periphery of the central cell in the wild type (Figure 4A), whereas they were not visible in *vks1* with a light microscope (Figure 4B). The two adjacent nuclei shown in Figure 4B likely originated from an abnormal nuclear division, as the lagging chromosomes were dispersed. In barley (*Hordeum vulgare*) and Arabidopsis, nuclei migrate to positions along the coenocytic membrane, where nuclear-cytoplasmic domains are formed by internuclear RMSs (Brown et al., 1994, 2003; Pickett-Heaps et al., 1999). As shown in Figure 4C, the nucleus was clearly surrounded by a domain where VKS1 and MTs were colocalized. In the absence of VKS1, the adjacent nuclei were embedded in a continuous MT network without a distinct interzone between them, thereby forming a flattened module closely adjacent to the cell membrane, and the nuclear-cytoplasmic domains was a primary casualty (Figure 4D). This finding may explain why no nuclei were clearly observed while they do exist in *vks1* with a light microscope, as shown in Figure 4B.

### VKS1 Is Essential for MT Organization in Mitosis and Cytokinesis

**Figure 8.**
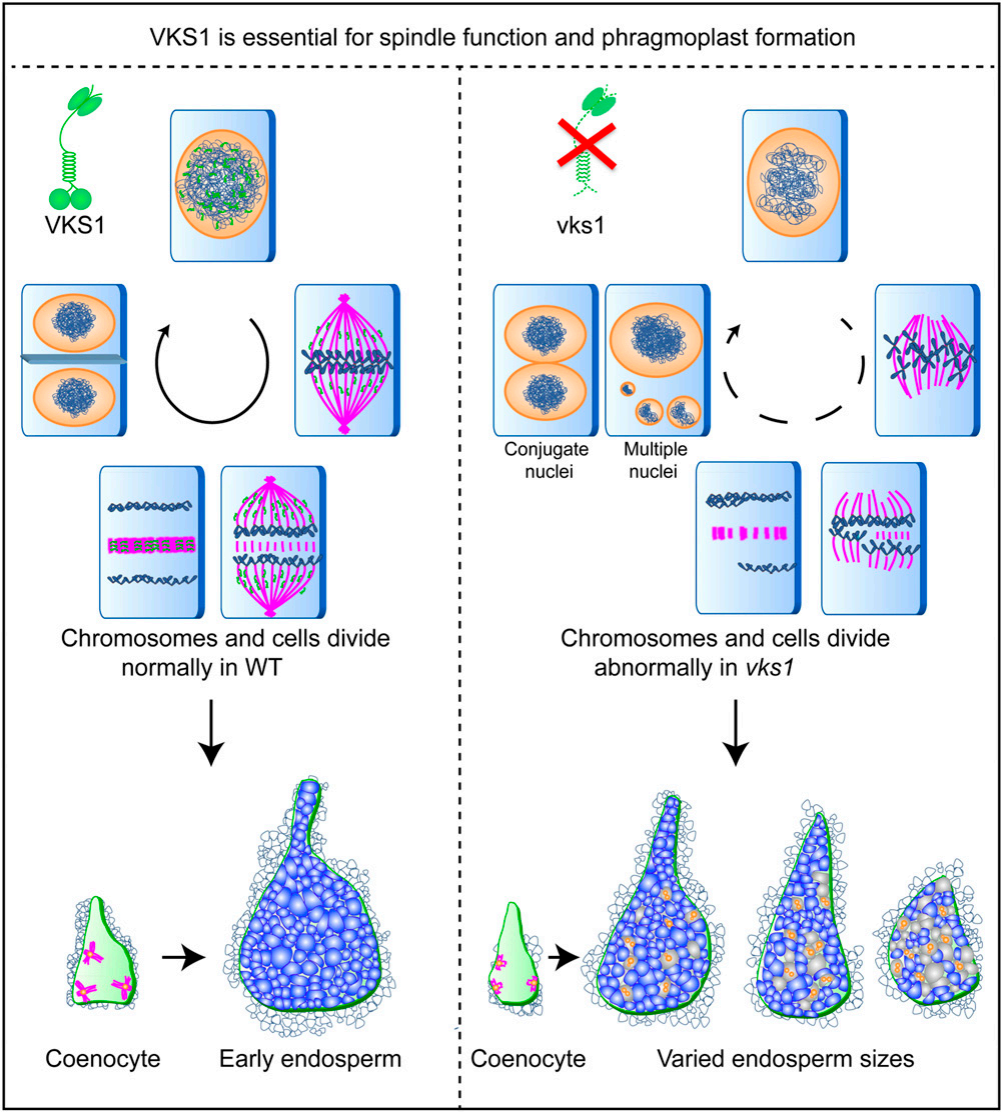
Proposed Model for VKS1 Function and the Varied-Kernel-Size Phenotype. Rapid mitotic divisions in early endosperm development produce most of the cells that make up the starchy endosperm. *Vks1* is predominantly expressed in early endosperm development and plays key roles in critical events of mitosis and cytokinesis. VKS1 dynamically colocalizes with MTs during interphase, mitosis, and cytokinesis. A lack of VKS1 results in substantial defects in spindle assembly, sister chromatid separation, phragmoplast formation, and eventually cell division. Therefore, while normal mitosis maintains rapid cell division to build up the endosperm structure and function in the wild type, aberrant mitosis may frequently cause repression in cell division, thereby leading to reduced cell numbers in the *vks1* endosperm. Perhaps as a consequence of varying numbers of affected cells, the kernel sizes of individual *vks1* seeds display dramatic variation.

Mitotic spindles are MT-based structures that function in the separation of sister chromatids during mitosis. An early step of spindle assembly involves nucleation of MTs. In most animals and fungi, centrosomes function as the major MTOCs to arrange MTs during both interphase and mitosis. Flowering plants lack centrosomes, and they assemble the spindle through a centrosome-free pathway (Bannigan et al., 2008; Yi and Goshima, 2018). During interphase, MTs are nucleated from the surface of the nuclear envelope and radiate into the cell, generating an array that resembles an aster, with the nucleus serving as the MTOC center. However, the factors and underlying mechanisms by which radial MTs are nucleated and formed remain largely unknown. In the *vks1* mutant, the number of radial MTs was obviously lower than that in the wild type (Figure 6A). Given that VKS1 and MTs also colocalize to the cytoplasm (Figure 3L), one could envision that VKS1 is essential for MT-mediated cytoplasmic MTOC formation. During prophase, cytoplasmic MTs are enriched around the nuclear envelope and later transform into bipolar structures called polar caps or the prophase spindles (De Mey et al., 1982; Zhang et al., 1990; Smirnova and Bajer, 1994, 1998; Chan et al., 2005). Interestingly, enriched cytoplasmic VKS1 proteins were observed to surround the nuclear envelope (Figure 5), suggesting that VKS1 is involved in the establishment of the prophase spindle. However, more detailed experiments are needed to test this hypothesis. A model for the main functions of VKS1 in mitosis and cytokinesis during early endosperm development is proposed, as shown in Figure 8. A lack of VKS1 causes substantial deformities in mitotic activities, including the missions of MTs to acquire chromosomes in prometaphase, spindle formation and chromosome alignment in metaphase, separation of sister chromatids in anaphase, and phragmoplast formation in telophase. In the *vks1* mutant, the assembly of MTs into a spindle was dramatically disrupted, resulting in a loose and splayed spindle (Figure 6; Supplemental Figure 9). This disruption caused incorrect alignment and separation of chromosomes in metaphase and anaphase. The multiple micronuclei observed in *vks1* cells could result from nondisjunction followed by nuclear restitution (Figures 7D and 7E). Therefore, while cell number is exponentially increased by rapid mitosis and cytokinesis in the wild-type endosperm, one could envision that cell division might be frequently repressed due to abnormal spindle formation.

The spindle is a dynamic structure that is precisely mediated by the balance of opposing forces provided by cross-linking motors (Desai and Mitchison, 1997; Heald, 2000). In some eukaryotes, cytoplasmic dynein serves as the primary cytoskeletal motor for minus-end-directed processes. A well-characterized example is the *Xenopus* system, in which disruption of dynein leads to splayed poles and abnormally long spindles (Heald et al., 1996). In addition, dynein functions to maintain the organization of the same polarity at spindle poles (Merdes et al., 2000). However, plants lack dynein but possess a large number of minus-ended kinesin-14s instead. These probably have evolved to function as dynein (Roberts et al., 2013). In addition to plants, Drosophila embryos possess C-terminal motors that function in pole formation via cross-linking the minus end (Compton, 1998). Previous studies demonstrated that a lack of kinesin-14 family members can lead to slower or less efficient spindle assembly (Matthies et al., 1996; Gordon et al., 2001; Prigozhina et al., 2001; Marcus et al., 2003). The Arabidopsis kinesin ATK5 specifically functions in early spindle assembly during mitosis; however, the spindle at metaphase in the *atk5* mutant showed no apparent phenotype (Ambrose and Cyr, 2007). Interestingly, the deformed spindle in *vks1* resembles the spindle fibers arrested in the first meiotic metaphase in Arabidopsis *atk1* and maize *dv1* (Chen et al., 2002; Ambrose and Cyr, 2007; Higgins et al., 2016). *Atk5* is the most closely related VKS1 homolog in Arabidopsis (Supplemental Figure 1).

Time-lapse imaging of mitosis revealed that lack of kinesin-14 (ATK5) caused mitosis to slow down (Ambrose and Cyr, 2007). During metaphase, we observed almost no cells in the dividing phase in a single field for the wild type. However, in *vks1*, cells at different phases of division were easily observed (Supplemental Figure 9), suggesting that mitosis in *vks1* slowed down, which in turn reduced early endosperm cell proliferation (Figure 8). In addition, our data also suggested that VKS1 plays a crucial role in spindle formation and chromosome separation in plants (Figure 6), which is consistent with prior studies on plant kinesin-14s (Chen et al., 2002; Ambrose and Cyr, 2007; Higgins et al., 2016; Dawe et al., 2018; Gicking et al., 2018). The CC domain in the VKS1 N terminus is responsible for localization to MTs (Figure 3H), and the motor in the C terminus generates opposing forces on the spindle MTs, thereby pulling the daughter chromosomes equally to opposite poles of the cell.

The *vks1* seeds exhibited varied kernel sizes within individuals. Consistently, *vks1* endosperms exhibited different severities of mitosis and cytokinesis abnormality at 6 DAP (Figures 7D and 7E). As early mitotic divisions are nonsynchronous, the timing at which aberrant mitosis occurs during proliferation of a clonal cell lineage and the number of cells that are affected at one time may differ within *vks1* endosperms. One could envision that mitotic abnormalities taking place earlier would produce a more severe phenotype than those taking place later.

Random chromosome loss may lead to PCD, and as a consequence there might be patches of death in the *vks1* endosperm. However, we did not observe any sector of cell death or cavity in the endosperm at different developmental stages (Figures 1H to 1L; Supplemental Figure 3B). This is probably because endosperm PCD is different from typical PCD programs in plants, wherein the cellular corpse is usually processed concurrently with cell death. In maize endosperm, PCD initiates in the central and upper regions of the starchy endosperm at 12 to 16 DAP, when the storage reserves are actively synthesized, and expands to the entire starchy endosperm by late development. The degradation of the cellular corpse does not occur until germination and is controlled exogenously by the activities of aleurone cells (Young and Gallie, 2000a, 2000b). It is likely that the processing of abnormal endosperm cells in *vks1* still follows the wild-type pattern.

Plant cells contain specialized MT-based structures called phragmoplasts, which facilitate cell wall formation following cell division. The phragmoplast contains a dynamic MT array that is aligned perpendicularly to the division plane, with the plus end locating to the division site. The absence of Arabidopsis kinesin-12A and kinesin-12B members caused a failure to form an antiparallel MT array and subsequently affected male gametogenesis without the formation of a cell plate (Lee et al., 2007). For kinesin 14, actions on the MT array of phragmoplasts, such as the sliding of antiparallel MTs, cross-linking of parallel MTs, and focusing of the spindle pole, require both the motor and tail domains of kinesin-14, of which the N-terminal tail attaches to one MT while the C-terminal motor transports another MT (Wendt et al., 2002; Ambrose et al., 2005; Fink et al., 2009; She and Yang, 2017). Loss of kinesin-14 may result in ineffective recruitment of MTs, leading to abnormal membrane formation, which in turn affects cell plate dynamics. Therefore, the defective phragmoplast in the *vks1* mutant may lead to failed cell wall formation at the cell plate and result in abnormal cells containing conjugate nuclei (Figures 7B, 7D, and 7E).

## METHODS

### Genetic Materials

All maize (*Zea mays*) plants were grown either in plastic greenhouses in Shanghai, China, or in the field in Sanyang and Harbin, China. The temperature in the greenhouses was 25 to 32°C during maize pollination and the early developmental stage (before 8 DAP). In addition to Shanghai, we also planted materials in Sanya, Hainan Province, for winter nursery and in Harbin, Heilongjiang Province, for summer nursery.

The pollen of the A619 inbred line was collected and mutagenized with EMS reagent for 45 min, and the resulting pollen was applied to A619 female ears. A large number of seeds were planted and self-pollinated, yielding more than 3000 ears, among which an ear segregated mutant seeds with varied kernel sizes; this was designated *vks1*. To purge most irrelevant mutations, *vks1* was recurrently backcrossed to A619 (used as the female) for six generations. Because *vks1* is a recessive mutation, the backcrossing and selfing of at least 20 plants in each generation had to be performed at the same time. The presence of the *vks1* allele in a plant was determined by inspecting whether the self-pollinated ear segregated *vks1* seeds. If it did, then the corresponding ear from backcrossing was used for planting in the next generation.

To knock out *Vks1* by CRISPR/Cas9, the small guide RNA was predicted online (http://www.e-crisp.org/E-CRISP/designcrispr.html) and synthesized by Sangon Biotech, then cloned into the *Psi*I and *Xba*I sites between the maize *U6* promoter and *U6* terminator. By *Agrobacterium tumefaciens*-mediated transformation, the constructs were delivered into maize. The inbred line B104 immature embryos (1.5–2.0 mm) were dissected from the ears growing in the field at ∼11 DAP. Three positive events were recovered, following the standard transformation protocol (Frame et al., 2002). The primers for making the construct and for amplifying the target region are listed in Supplemental Table 1.

### Mapping by Sequencing of *vks1*

To map the *vks1* mutant gene, *vks1* and wild-type A619 plants were crossed and then self-pollinated. More than 100 seeds of each phenotype (mutant and normal) from the segregating ears were selected and germinated individually for DNA extraction. For each pool, DNA from each sample was mixed in an equal ratio. Genomic DNA was sheared for preparation of the sequencing library according to the standard protocol of the Illumina TruSeq DNA PCR-free prep kit.

Reads of the two bulks (A, normal, and B, *vks1*) and the parental line A619 were aligned to the reference genome using bwa software with default parameters; SNP calling was performed using GATK software. SNPs with a sequencing depth of <5 were filtered. Then, the SNP index was calculated, and SNPs with an SNP index of <0.3 in both bulks were filtered to reduced false-positive detection of SNPs. The MutMap method was applied to detect genomic intervals underlying the phenotype as described previously (Takagi et al., 2013). Briefly, the average SNP index was calculated using a sliding-window approach with a 4-Mb window size and a 200-kb increment. Δ (SNP index), the difference between the two bulks, was then determined. Statistical confidence intervals of Δ (SNP index) were calculated under the null hypothesis of no quantitative trait loci following procedures reported previously (Abe et al., 2012; Takagi et al., 2013).

Fine-mapping of *vks1* was performed using the BC1F1 population of *vks1* × (*vks1* × B73). Insertion/deletion primers (shown in Supplement Table 1) were based on differences in B73 and A619 genomic sequences on chromosome 7. Linkage analysis of the BC1F1 population was performed by detecting the genotype of the reference mutation site using the primers vks1-F and vks1-R. The splice acceptor site of *vks1-2* sites was detected by the primers spc-F and spc-R, and *Mu* insertion in the 5′UTRwas detected by the primers F, R, and TIR6, as shown in Supplemental Figure 5.

### Phenotypic Analysis

One hundred seeds in the middle of the ear were selected for measurement of single-grain weight. All the ears used for photography were representative of more than 10 ears. Kernels were selected from the middle of the ear for photography and determination of single-grain weight. The coefficient of variation (i.e., the ratio of the sd to the mean [CV = σ/μ]), was calculated. Plants grown in the field for 10 weeks were transferred to flowerpots for photography. After the plants were photographed, the leaves were removed, and the whole plants were photographed again.

### Measurement of Proteins and Starch

To measure the content of the proteins and starch, 20 mature kernels of A619 and *vks1* from three individual ears were ground into fine flour using steel beads. Then, 100 mg of flour from each sample was used for the measurement of proteins and starch. Three biological replicates for each were performed. SDS-PAGE was performed to analyze the accumulation pattern of zein and nonzein proteins in A619 and *vks1* according to previous reports (Liu et al., 2016; Zhang et al., 2016). The starch content was detected using the Total Starch Assay Kit (K-TSTA; Megazyme).

### Pollen Viability

Fresh pollen from dehiscent tassels of A619 and *vks1* was collected on slides and then stained with a solution containing 0.2% (w/v) benzidine, 0.2% (w/v) α-naphthol, and 0.3% (v/v) H_2_O_2_ for 5 min. Slides were viewed with a light microscope (Leica M165 FC). Ten tassels of A619 and *vks1* were detected. DeepRed-stained pollen grains were fertile, whereas irregularly shaped, unstained grains were sterile. Then, the ratio of the number of dark red pollen grains to the total number of pollen grains in the microscope field of A619 or *vks1* was calculated. The average value of the ratios of 10 fields was used to indicate pollen fertility.

### Histocytochemical Analysis

For the histocytochemical analysis of endosperm tissues of A619 and *vks1*, seeds at the early developmental stage (2, 4, and 6 DAP) and filling stage (18 DAP) were collected. Seeds were fixed in FAA buffer (50% ethanol:formaldehyde:acetic acid = 90:5:5 volume) and then vacuumed at 4°C. After dehydration using a gradient concentration of ethanol, 2-, 4-, and 6-DAP-old seeds were embedded in epoxide resin for semithin sectioning, while 18-DAP-old samples were embedded in paraffin. The sections were stained with toluidine blue solution and then photographed under a bright field using an ECLIPSE 80i microscope (Nikon).

To calculate the number of cells in the early (4 and 6 DAP) endosperm of A619 and the mutant *vks1*, 20 semithin sections were imported into Adobe Photoshop software, and the number of cells was determined by cell counting. Then the average cell number of 20 sections was calculated.

To calculate the area of the endosperm relative to that of the whole seed, the area of the endosperm and the area of the whole seed of 20 sections were measured by ImageJ software. Then, the ratio of endosperm area to seed area was calculated to indicate the relative value.

### RT-qPCR and Standard RT-PCR

Total RNA was extracted from whole seeds or endosperms of A619 and *vks1* at 0, 1, 2, 3, 4, 6, 8, 10, 12, 16, 18, and 24 DAP using TRIzol reagent (Invitrogen, catalog number 15,596,018). Then, RNA was purified with an RNeasy Mini Kit (Qiagen, catalog number 74,106) after DNaseI digestion (Qiagen, catalog number 79,254) for reverse transcription by a SuperScript III First Strand Synthesis Kit (Invitrogen, catalog number 18,080,051). RT-qPCR was performed with SYBR Green I (Takara, catalog number RR420A) and a CFX Connect Real-Time System (Bio-Rad). The primers of *Vks1* were Vks1-RTF and Vks1-RTR. The comparative CT method (ΔΔCT method) was employed for the relative quantification of gene expression, and the maize *Actin* gene (primers: Actin-RTF and Actin-RTR) was used as a control. The expression level in seeds at 0 DAP was set to 1.

To analyze the expression level of *Vks1* in *vks1-2*, RNA was extracted from the 2-DAP seeds of B73 and *vks1-2* and used for RT-qPCR analysis. The gene-specific primers for *Vks1* and *Actin* were shown above. The expression level in B73 seeds at 2 DAP was set to 1.

Data were generated from three biological replicates of each sample, and means and se were calculated using Microsoft Excel.

### RNA in Situ Hybridization

To analyze the tissue expression pattern of *Vks1* in the seeds at early stages, 2- and 3-DAP-old seeds of A619 were used for RNA in situ hybridization as described in our previous work (Zhang et al., 2015). The seeds were fixed for 16 h in 4% paraformaldehyde solution with 0.1% Triton X-100 and 0.1% Tween 20 in PBS (Takara, catalog number T900). After dehydration using graded ethanol and vitrification by dimethylbenzene, the samples were embedded in paraffin. A 524-bp cDNA fragment of *Vks1* was cloned using the primers Vks1-probeF*Kpn*I and Vks1-probeR*Xho*I and inserted into the pBluescript SK+ vector using *Kpn*I and *Xho*I. The vector was used for the synthesis of antisense and sense RNA probes according to the instructions for the DIG RNA Labeling Mixture (Roche, catalog number 11175025910). Then, 10-μm sections were cut, and in situ hybridization experiments were performed according to methods described previously (Langdale, 1994; Cox and Goldberg, 1998). The sections were observed and photographed with an ECLIPSE 80i microscope.

### Anti-VKS1 Antibody Preparation

VKS1 antibodies were produced by ABclonal. The cDNA fragment encoding the partial VKS1 protein from amino acids 49 to 449 was amplified using the primers VKS1-EF and VKS1-ER and then inserted into the prokaryotic expression vector pET-28a-VKS1 using MultiF Seamless Assembly Mix (ABclonal, catalog number RK21020). Purified protein was injected into rabbits to produce VKS1 antibodies. After affinity purification, the antibodies were used for protein gel blotting and immunofluorescence histochemistry.

### Immunoblotting Analysis

Total protein from 2- and 3-DAP seeds of A619 and *vks1* was extracted with radioimmunoprecipitation assay lysis buffer (Leagene, catalog number PS0013). Proteins were quantified by Bradford assays. Twenty micrograms of protein was separated by a 10% SDS-PAGE gel and then transferred electrophoretically to a PVDF membrane (Bio-Rad, catalog number 162-0177). The membrane was incubated with VKS1 antibodies at 4°C overnight and then incubated with the secondary antibody, goat anti-rabbit IgG-horseradish peroxidase (HRP; Abmart, catalog number M21002L). To detect the control protein ACTIN, a primary antibody, mouse monoclonal ACTIN antibody (Abmart, catalog number M20009L), and a secondary antibody, goat anti-mouse IgG-HRP (Abmart, catalog number M21001L), were used to perform the immune reaction. The dilution of antibodies was 1:1000. Finally, the membrane was treated with HRP chemiluminescent substrate reagent (Invitrogen, catalog number WP20005) and imaged using a Tanon-5200 system (Tanon).

To analyze the protein content of VKS1 in *vks1-2*, protein was extracted from the 2-DAP seeds of B73 and *vks1-2*.

### Subcellular Localization of VKS1 in *Nicotiana benthamiana* Epidermal Cells

To investigate the subcellular localization of VKS1 in *N. benthamiana* leaves, the full-length and different domain fragments of VKS1 protein were fused with GFP in pCAMBIA1301 plasmid using *Kpn*I and *Xho*I and expressed via the 35S promoter. The primer pairs (shown in Supplemental Table 1) VKS1^FL^-F*Kpn*I and VKS1^FL^-R*Xho*I for the full-length VKS1 protein (VKS1^FL^, amino acids 1–761), VKS1^△T-^F*Kpn*I and VKS1^FL^-R*Xho*I for the VKS1 protein lacking the tail domain (VKS1^△T^, amino acids 49–761), VKS1^FL^-F1*Kpn*I and VKS1^△M^-R*Xho*I for the VKS1 protein lacking the motor (VKS1^△M^, amino acids 1–408), VKS1^△T^-F*Kpn*I and VKS1^△M^-R*Xho*I for the VKS1 protein lacking the tail and motor domains (VKS1^△TM^, amino acids 49–408), and VKS1^△TCC^-F*Kpn*I and VKS1^FL^-R*Xho*I for the VKS1 protein lacking the tail and CC domains (VKS1^△TCC^, amino acids 409–761) were used to amplify the different fragments of VKS1. In addition, GFP-VKS1^△TCC^-F and GFP-VKS1^△TCC^-R were used to amplify the motor domain, which was ligated to the C terminus of GFP by the homologous recombination method. These constructs were then transferred into *Agrobacterium* (GV3101 strain) and injected into 3-week-old *N. benthamiana* leaves. To analyze the colocalization of VKS1 with MTs, the *Agrobacterium* containing the Arabidopsis β-tubulin6 fused with the mCherry protein (mCherry-TUB6) was infiltrated into *N. benthamiana* leaves together with the *Agrobacterium* carrying VKS1-GFP.

Two days after infiltration, the transfected leaves were collected for imaging. To observe the nucleus, 1 μg/mL DAPI (Roche, catalog number 0671) solution was injected into the transfected leaves. Afterwards, segments of transfected leaf were observed with an LSM880 confocal microscope (Zeiss). Z-stack images of epidermal cells expressing GFP-fused protein were taken in the Airyscan superresolution mode.

### Fluorescence Immunohistochemistry Analysis

For immunolocalization of tubulin in endosperm cells during mitosis, seeds of A619 and *vks1* were used at 6 DAP. The sample treatment was performed according to a previous report (Quan et al., 2008). Hand sections of seeds were fixed for 1 h in freshly prepared 4% (v/v) formaldehyde in PHEM buffer with 0.02% (v/v) Triton X-100 and 5% (v/v) DMSO. After being rinsed several times in PHEM buffer, the samples were treated with a cell wall-removal enzyme solution containing 1% (w/v) β-glucuronidase (LabTop, catalog number S10046), 0.1% (w/v) cellulose (Yakult, catalog number 9012-54-8), 0.1% (w/v) pectinase (LabTop, catalog number S10007), 0.1% (w/v) lyticase (Solarbio, catalog number L8141), and 1% (w/v) Glc for 30 min. Samples were then blocked with 5% (w/v) BSA (Yeasen, catalog number 36103ES60) in PBS for 1 h and hybridized with monoclonal mouse anti-α-tubulin antibodies (1:100; Sigma-Aldrich, catalog number T6074) overnight in the dark at 4°C. After being washed three times with PBS, the samples were incubated with the secondary antibody Alexa Fluor 488-conjugated goat anti-mouse IgG at a dilution of 1:200 (Yeasen, catalog number 33206ES60). To analyze the localization of VKS1 during mitosis, the blocked sections were incubated in VKS1 antibodies and then hybridized with Alexa Fluor 488-conjugated goat anti-rabbit IgG secondary antibody (Yeasen, catalog number 33106ES60).

To detect the colocalization of VKS1 and tubulin in the endosperm cells undergoing mitosis, seeds at 2 and 6 DAP were fixed in fixative buffer (4% [v/v] paraformaldehyde, 0.1% [v/v] Triton X-100, and 0.1% [v/v] Tween 20 in PBS, pH 7.0) for 16 h and then embedded in paraffin. After being blocked in 5% (w/v) BSA, the sections were coincubated with anti-VKS1 antibodies raised in rabbit and α-tubulin antibodies raised in mouse and then stained by Alexa Fluor 488-conjugated goat anti-rabbit and Alexa Fluor 594-conjugated goat anti-mouse IgG (Yeasen, catalog number 33212ES60) secondary antibodies, respectively.

Nuclei were stained with 1 μg/mL DAPI. The sections were observed with an LSM880 confocal microscope under Airyscan modes (Zeiss). Z-stack images of endosperm cells undergoing mitosis and images of nuclei of endosperm cells were taken in the Airyscan superresolution mode.

### Phylogenetic Analysis

A total of 163 kinesin protein sequences were submitted to the ClustalW program using the default settings (pairwise alignment options: gap opening penalty 10, gap extension penalty 0.1; multiple alignment options: gap opening penalty 10, gap extension penalty 0.2, gap distance 4, no end gaps and protein weight matrix using Gonnet) for multiple protein alignment. Based on the aligned protein sequences, the phylogenetic tree was constructed using the MEGA7.0 program (http://www.megasoftware.net/) and the maximum likelihood method with the Jones-Taylor-Thornton model, and the bootstrap test was conducted with 1000 replicates.

### Accession Numbers

Sequence data from this study can be found in GenBank/EMBL data libraries under accession numbers NP_001292799 for VKS1 (ZmKIN11) and NP_001306694 for DV1. The other accession numbers used for phylogenetic analysis can be found in Supplemental Fileand Supplemental Figure 6.

### Supplemental Data

**Supplemental Figure 1.** Phylogenetic analysis of the kinesin family.
**Supplemental Figure 2.** Investigation of maternal effect on the *vks1* phenotype.
**Supplemental Figure 3.** Storage reserve accumulation and plant phenotype of A619 and *vks1*.
**Supplemental Figure 4.** Segregation of mutant and normal seeds on F2 ears of A619 and *vks1*.
**Supplemental Figure 5.** Map-based cloning of *vks1* and the kernel of *vks1-3* and *vks1-4*.
**Supplemental Figure 6.** Amino acid sequence alignment of *Vks1* and its homologs in other grasses.
**Supplemental Figure 7.** Immunofluorescence histochemical localization of VKS1 and α-tubulin in A619 endosperm cells.
**Supplemental Figure 8.** Dual immunofluorescence localization of VKS1 and α-tubulin in telophase of endosperm cells.
**Supplemental Figure 9.** Immunofluorescence histochemical analysis of defective mitosis in *vks1* endosperm cells.
**Supplemental Figure 10.** Expression pattern of the maize kinesin family.
**Supplemental Table 1.** Primers used in this study.
**Supplemental File.** Text file of the alignment used to generate the phylogenetic tree in Supplemental Figure 1.

## Dive Curated Terms

The following phenotypic, genotypic, and functional terms are of significance to the work described in this paper:

ATK5 Gramene: AT4G05190
ATK5 Araport: AT4G05190

ATK1 Gramene: AT4G21270
ATK1 Araport: AT4G21270

IKU2 Gramene: AT3G19700
IKU2 Araport: AT3G19700

DAPI CHEBI: CHEBI:51231
